# Probiotics intervention in preventing conversion of impaired glucose tolerance to diabetes: The PPDP follow-on study

**DOI:** 10.3389/fendo.2023.1113611

**Published:** 2023-02-17

**Authors:** Qun Yan, Weiting Hu, Yan Tian, Xu Li, Yuan Yu, Xing Li, Bo Feng

**Affiliations:** ^1^ Department of Endocrinology, Shanghai East Hospital, Tongji University School of Medicine, Shanghai, China; ^2^ The Second Clinical Medical College, Shanxi Medical University, Second Hospital of Shanxi Medical University, Taiyuan, China

**Keywords:** probiotic, impaired glucose tolerance, diabetes mellitus, type 2, gut microbiota, conversion

## Abstract

**Objectives:**

The purpose of this study was to assess the incidence of type 2 diabetes mellitus (T2DM) after 6 years in patients with IGT who received early probiotic intervention in the Probiotics Prevention Diabetes Program (PPDP) trial.

**Methods:**

77 patients with IGT in the PPDP trial were randomized to either probiotic or placebo. After the completion of the trial, 39 non-T2DM patients were invited to follow up glucose metabolism after the next 4 years. The incidence of T2DM in each group was assessed using Kaplan-Meier analysis. The 16S rDNA sequencing technology was used to analyze gut microbiota’s structural composition and abundance changes between the groups.

**Results:**

The cumulative incidence of T2DM was 59.1% with probiotic treatment versus 54.5% with placebo within 6 years, there was no significant difference in the risk of developing T2DM between the two groups (*P*=0.674).

**Conclusions:**

Supplemental probiotic therapy does not reduce the risk of IGT conversion to T2DM.

**Clinical Trial Registration:**

https://www.chictr.org.cn/showproj.aspx?proj=5543, identifier ChiCTR-TRC-13004024.

## 1 Introduction

Compared with normal glucose tolerance (NGT), people with prediabetes, especially impaired glucose tolerance (IGT), have a higher risk of developing type 2 diabetes mellitus (T2DM). Early intervention can significantly reduce the probability of developing T2DM in the IGT population (1–3). The dysbiosis in the gut microbiota has recently been recognized as a critical environmental factor in individuals with prediabetes or diabetes mellitus (DM) (4, 5). Several studies (6–8) showed that probiotic and synbiotic intake affects the glycemic profile in patients with prediabetes and T2DM. Most recently, the Probiotics Prevention Diabetes Program (PPDP) Study demonstrated that probiotic supplementation during two years did not improve fasting plasma glucose(FPG) levels and did not reduce the risk of conversion of IGT to T2DM (9).

After the PPDP trial was completed, participants without T2DM were invited to follow up for 4 years. The objective of the PPDP Follow-On study was to observe the effect of early probiotic intervention on the conversion of T2DM after 6 years.

## 2 Research design and methods

### 2.1 PPDP study

The design and primary results of the PPDP study have been reported previously (9, 10). Briefly, the PPDP Study included 77 patients diagnosed with IGT in the outpatient department of Shanghai East Hospital of Tongji University from September 2014 to September 2016. IGT and T2DM were diagnosed according to the 1999 WHO Criteria. IGT was diagnosed when FPG <7.0 mmol/L, and oral glucose tolerance test (OGTT): 2-h post glucose load ≥ 7.8 and <11.0 mmol/L. T2DM was diagnosed when FPG ≥7.0 mmol/L and or OGTT: 2-h post glucose load≥11.1 mmol/L, or self-reported diabetes history and being treated with hypoglycemic agents. All participants were randomized, double-blind to receive probiotics (including Bifidobacterium, Lactobacillus acidophilus and Enterococcus faecalis) or matched placebo. Probiotics were produced by Shanghai Sine Pharmaceutical Laboratories Co, Ltd. For both groups, the doses were 840 mg daily, 210 mg per one pill, two pills per time, and two times daily. Both groups were followed for two years with an OGTT every 3 months in the first year and every 4 months in the second year to assess the patient’s glucose metabolism. At the end of the PPDP study, there were 20 patients in the Probiotics group and 13 in the Placebo group who developed T2DM.

Feces of the two groups before and after intervention were collected. The 16S rDNA sequencing technology was used to analyze intestinal microbiota’s structural composition and abundance changes. The primary outcome was the cumulative prevalence of T2DM in the two groups. The secondary endpoints were the possible changes in the proportion of microbiota. The study was registered in the Chinese clinical trial registry (ChiCTR-TRC-13004024).

### 2.2 PPDP follow-on study

**Figure 1:**
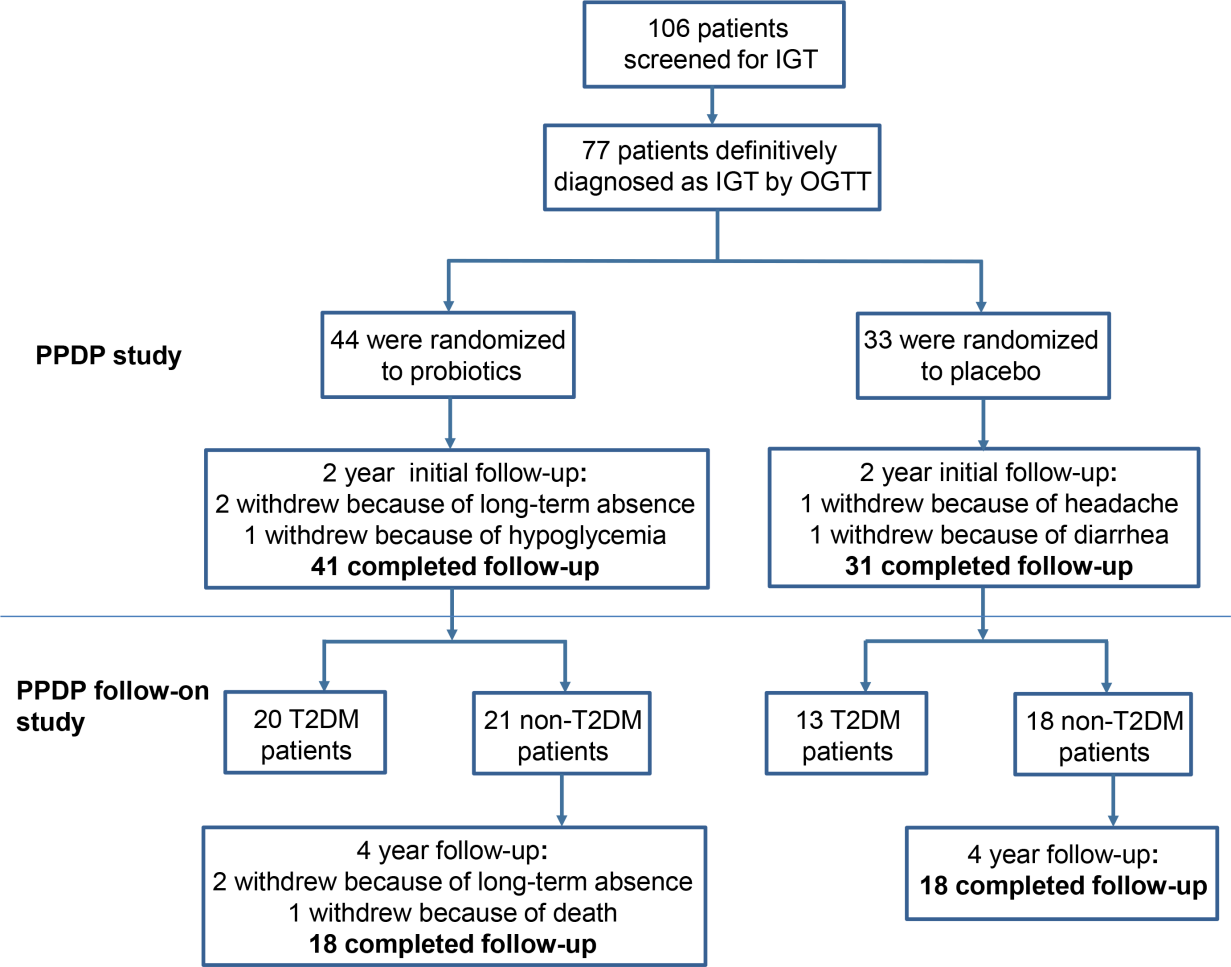
Flow diagram for PPDP follow-on study.

After the completion of the initial PPDP study, patients who with undiagnosed T2DM continue to be invited to participate in the PPDP Follow-On study without probiotics intervention. A total of 39 non-T2DM patients agreed to follow up glucose metabolism for next 4 years. Patients were asked to monitor fasting and postprandial blood glucose by themselves. At the 4th year, OGTT were assessed at the outpatient department of Shanghai East Hospital. Finally, 36 patients finished the next 4-year follow-up, 2 patients withdrew due to loss of contact, and 1 patient died due to a blood tumor, with a dropout rate of 4.2%. The detailed patient flow of the original trial (PPDP) and follow-on study is summarized in **Figure 1**.

The primary outcome was the cumulative incidence of T2DM in the two groups during the 6 years. The PPDP study and the PPDP Follow-On study were reviewed and approved by the hospital’s ethics committee and all patients signed informed consent.

### 2.3 16S rRNA gene sequencing and analysis

Fresh fecal samples were collected and bacteria’s 16S rRNA gene sequence was detected using paired-end configuration on an Illumina MiSeq system (Illumina, San Diego, USA). Briefly, microbial DNA was extracted and DNA quality was examined by agarose gel electrophoresis. The V3-V4 regions of the bacteria’s 16S rRNA gene were amplified by PCR. The sequencing was performed using paired-end configuration on an Illumina MiSeq system (Illumina, San Diego, USA). Raw fastq files were demultiplexed, and then data was filtered to ensure quality. The taxonomy of each 16S rRNA gene sequence was analyzed by RDP Classifier (http://rdp.cme.msu.edu/) against the silva (SSU115)16S rRNA database. The detail of sequencing and analysis showed in **Supplementary Material 1**.

### 2.4 Statistical analyses

Statistical analyses were performed by SPSS version 23.0 (IBM Corp, Armonk, NY, USA) and GraphPad Prism version 8.0 (San Diego, California, USA). Continuous data were described as means ± standard deviation, and inter-group comparison was performed with a t-test or analysis of variance. All continuous data were abnormally distributed. Categorical data were described as n (%), and inter-group comparisons were analyzed by χ^2^ -test. Mann–Whitney test was used to compare data that were not normally distributed between the groups. The cumulative incidence of T2DM = (the number of cases developing T2DM after 6 years of follow-up/the number of cases starting follow-up) × 100%. The difference in the incidence of T2DM between the probiotic group and the placebo group over time was analyzed using the Kaplan-Meier survival curve. COX regression analysis was used to analyze the influencing factors of T2DM. The risk was described as Hazard ratio (HR) and 95%CI. P<0.05 was statistically significant.

## 3 Results

### 3.1 Baseline characteristics

The baseline characteristics of the Probiotics group and Placebo group in the PPDP study have been presented in the previous article (9). Specifically, there were no significant differences in sex composition, age, body mass index (BMI), blood pressure, heart rate, liver function, blood lipid profile, FPG, post-glucose load plasma glucose, glycated hemoglobin A1c(HbA1c), fasting serum insulin (FINS) and the homeostasis model assessment of insulin resistance (HOMA-IR) between the two groups.

At the end of the PPDP study, there remained 39 patients with undiagnosed T2DM (21 patients in the Probiotics group and 18 in the Placebo group). The characteristics of undiagnosed T2DM patients at the end of 2-year follow up between the probiotics and placebo groups were shown in **Table 1**.

**Table 1:** Characteristics of undiagnosed T2DM patients at the end of 2-year follow up between the Probiotics and Placebo groups.

	Probiotics group (n=21)	Placebo group (n=18)	*P* value
Age (year)	62.3 ± 10.2	52.1 ± 14.7	0.015*
Male n(%)	7 (33.3)	9 (50.0)	0.342
BMI (kg/m^2^)	25.2 ± 2.3	24.3 ± 2.3	0.483
WC (cm)	88.2 ± 7.5	87.8 ± 11.9	0.903
SBP (mmHg)	125.3 ± 12.3	119.3 ± 18.3	0.270
DBP (mmHg)	79.1 ± 9.4	76.1 ± 8.7	0.348
ALT (IU/L)	24.8 ± 15.8	23.4 ± 23.3	0.851
SCr (umol/L)	64.8 ± 9.4	62.1 ± 12.0	0.690
TG (mmol/L)	1.2 ± 0.59	1.69 ± 1.09	0.181
TC (mmol/L)	4.51 ± 0.99	4.99 ± 1.01	0.157
HDL-C(mmol/L)	1.51 ± 0.30	1.43 ± 0.48	0.555
LDL-C (mmol/L)	2.91 ± 0.91	3.22 ± 0.97	0.212
FPG (mmol/L)	5.25 ± 0.47	5.23 ± 9.54	0.933
30minPG (mmol/L)	9.72 ± 1.62	9.40 ± 1.79	0.604
2hPG (mmol/L)	7.76 ± 1.25	7.23 ± 0.88	0.154
FINS (μU/L)	11.7 ± 7.9	11.1 ± 10.8	0.850
30minINS (μU/L)	77.8 ± 53.8	75.5 ± 61.1	0.906
2hINS (μU/L)	99.2 ± 64.4	64.4 ± 47.4	0.075
HOMA-IR	2.79 ± 2.07	2.59 ± 2.45	0.802
Incidence of T2DM 4 years later (n)	6	5	–

ALT, Alanine aminotransferase; BMI, body mass index; DBP, Diastolic blood pressure; FPG, fasting plasma glucose; FINS, fasting insulin; HOMA-IR, homeostasis model assessment of insulin resistance; HDL-C, high-density lipoprotein cholesterol; LDL-C, low-density lipoprotein cholesterol; SBP, systolic blood pressure; TC, total cholesterol; TG, Triglyceride; SCr, serum creatinine; 30minPG, 30-minute-plasma glucose post-glucose load; 2hPG, 2-h-plasma glucose post-glucose load; 30mINS, 30-minute-insulin post-glucose load; 2hINS, 2-hour-insulin post-glucose load.

Categorical data were described as n (%). Continuous data are presented as mean ± SD.

*P values<0.05 were considered significant.

### 3.2 Comparison of the incidence of T2DM

**Figure 2:**
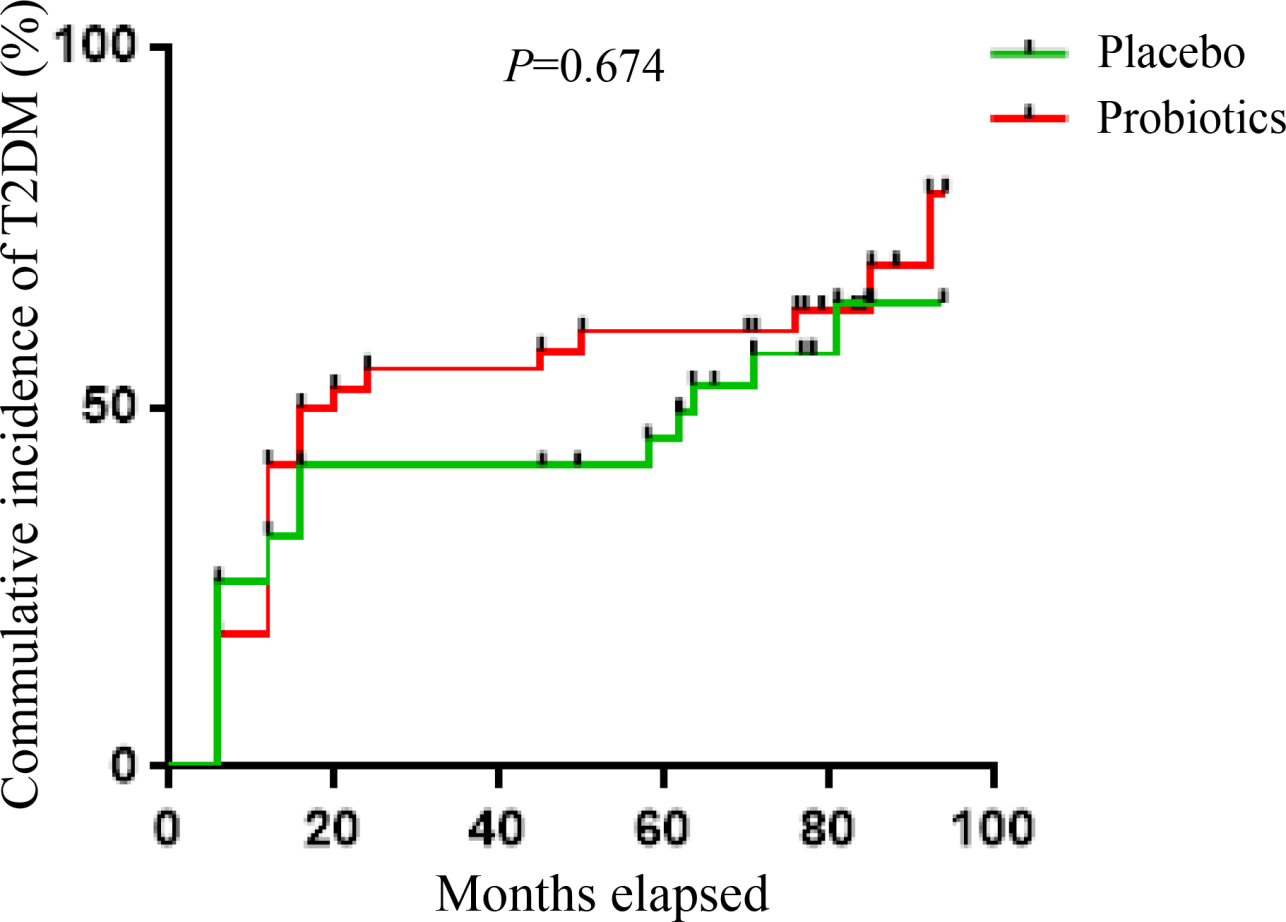
Kaplan-Meier analysis of the cumulative incidence of T2DM within 6 years between the probiotics and placebo group.

In the next 4 years, there were 6 patients in the Probiotics group and 5 patients in the Placebo group who developed T2DM. Thus, the cumulative incidence of T2DM was 59.1% in the probiotic group and 54.5% in the placebo group within 6 years. As shown in **Figure 2**, there was no significant difference in the risk of developing T2DM between the two groups within 6 years (*P*=0.674).

At the end of the 6-year follow-up, patients were grouped according to whether T2DM occurred. The age of the T2DM group was significantly older than the non-T2DM group (57.2 vs 53.7 years, *P*=0.004). There was no significant difference in other clinical data between the two groups (all *P >*0.05).

### 3.3 COX regression analysis of risk factors for T2DM

**Figure 3:**
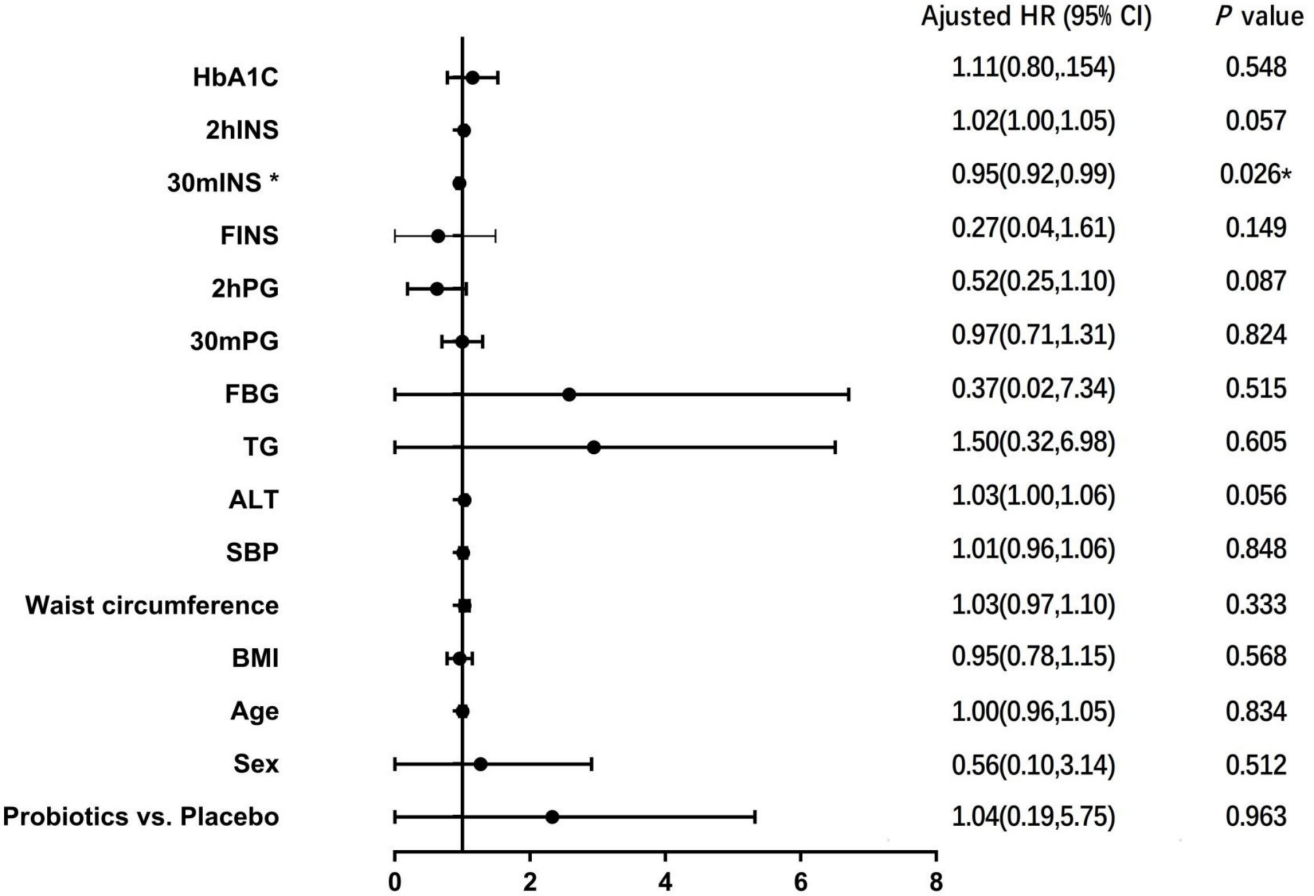
COX regression analysis of risk factors for T2DM after 6 years.

COX regression model was used to analyze the risk factors affecting the development of T2DM. Probiotic intervention or not, age, gender, BMI, waist circumference, blood pressure, liver function, blood lipid, blood glucose, serum insulin and HbA1c were used as covariates, and results showed that 30-minute post-glucose load insulin level was a factor affecting the conversion of IGT to T2DM (HR=0.954, 95%CI 0.915-0.994, *P* =0.026) (**Figure 3**).

### 3.4 Gut microbiota analysis

**Figure 4:**
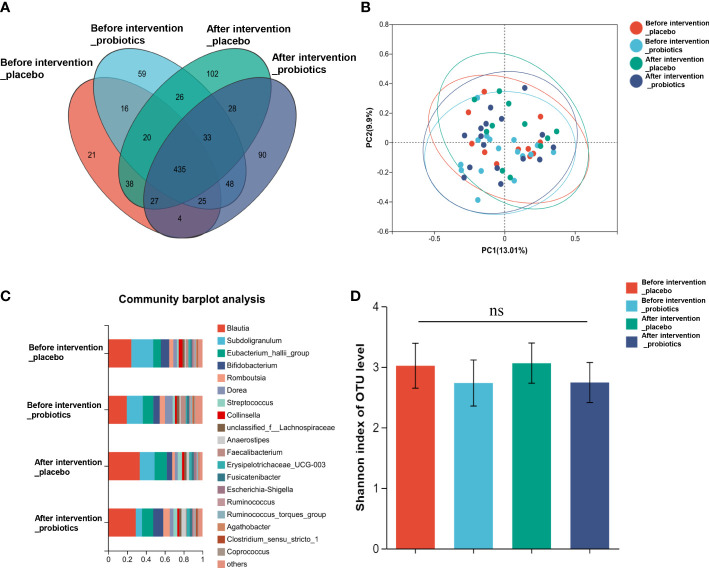
Composition and diversity of gut microbiota before and after two years probiotics or placebo intervention. **(A)** The Venn diagram shows the common or endemic species between groups in the level of OUT; **(B)** Weighted UniFrac PCoA; **(C)** Compositional change at the genus level; **(D)** α diversity analysis of gut microbiota.

**Figure 5:**
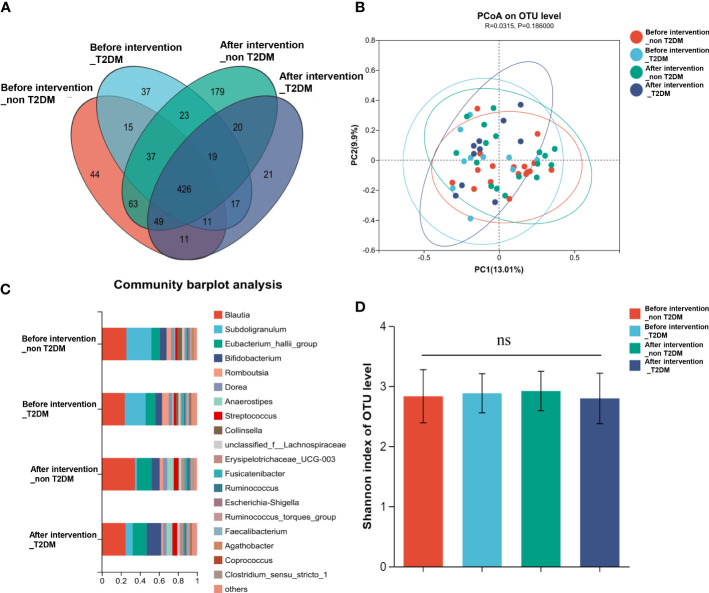
Composition and diversity of gut microbiota in T2DM patients and non-T2DM patients before and after two years of probiotics intervention. **(A)** The Venn diagram shows the common or endemic species between groups in the level of OUT; **(B)** Weighted UniFrac PCoA; **(C)** Compositional change at the genus level; **(D)** α diversity analysis of gut microbiota.

According to the informed consent and research protocol, fecal samples were collected at baseline (day 0) and the end of the 2-year follow-up visit. The 16S rDNA sequencing technology was used to analyze gut microbiota’s structural composition and abundance changes. A total of 32 stool samples in the Probiotic group and 22 in the Placebo group were collected. In this study, the differences in operational taxonomic units (OTUs) abundance among the probiotic group and the placebo group were compared. The Venn diagram showed that 435 of the total 972 genera were shared among the 4 groups (**Figure 4A**). To display microbiome space between samples, principal coordinates analysis (PCoA) was performed. The results showed that the microbiota was similar between the probiotic and placebo interventions (**Figure 4B**), and the probiotic intervention might not have caused the recombination of the microbial community composition. Microbial community variation was also analyzed. At the genus level, Blautia, Subdoligranulum, Eubacterium hallii, Bifidobacterium, and Romboutsia accounted for the majority in each group (**Figure 4C**). However, there were no statistically significant changes in the microbiota composition after probiotics or placebos intervention in any of the groups compared with those before intervention. To assess the differences in bacterial diversity among groups, sequences were aligned for alpha-diversity. No significant difference in the Shannon index between the probiotic group and the placebo group was observed. (**Figure 4D**). We also compared the difference in gut microbiota’s structural composition and abundance changes between the baseline and after intervention among T2DM and non-T2DM groups. The Venn diagram of bacteria showed that 426 of the total 972 genera were shared among the 4 groups (**Figure 5A**). The results of PCoA showed that the microbiota was similar between the T2DM group and the non-T2DM group both at baseline and after intervention (**Figure 5B**). Microbial community analysis demonstrated that Blautia, Subdoligranulum, Eubacterium hallii, Bifidobacterium, and Romboutsia accounted for the majority in each group (**Figure 5C**). At baseline and after the intervention, no significant difference in the Shannon index between the T2DM group and the non-T2DM group was observed. (**Figure 5D**).

**Figure 6:**
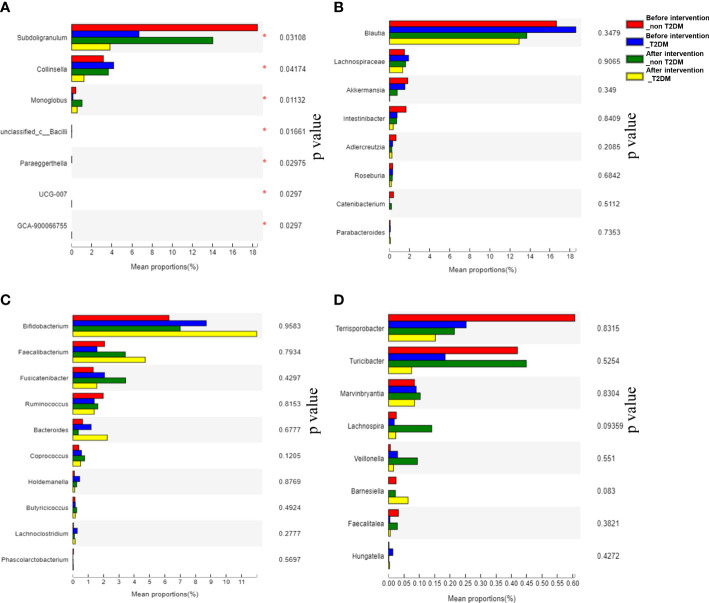
Analysis of significantly altered gut microbiota and specific species microbiot in T2DM patients and non-T2DM patients after two years of probiotics intervention. **(A)** Analysis of significantly altered gut microbiota; **(B)** Analysis of metabolism-related microbiota; **(C)** Analysis of producing short-chain fatty acids-related microbiota; **(D)** Analysis of gut probiotics.

**Figure 7:**
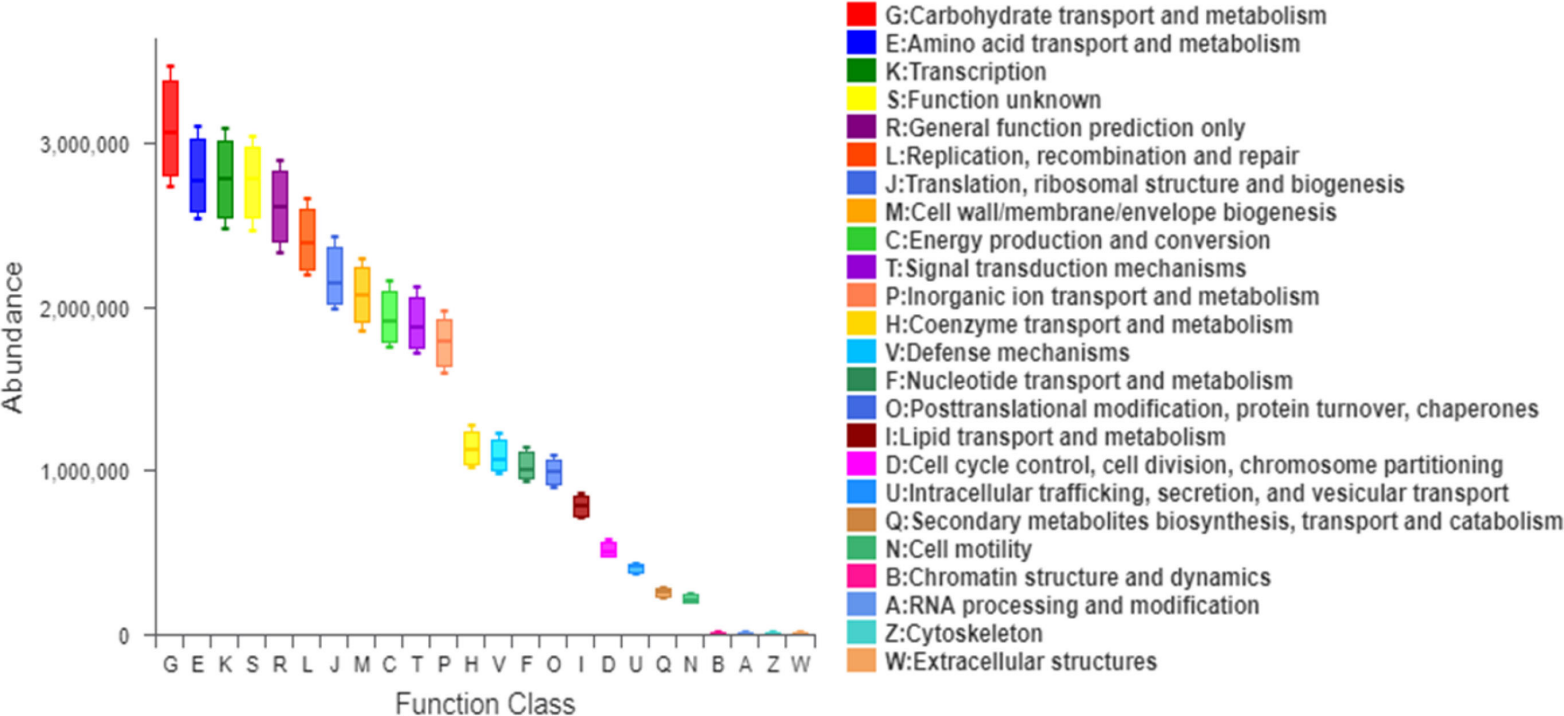
Functional prediction and comparison of gut microbiota between T2DM and non-T2DM groups.

The mean proportion of subdoligranulum and monoglobus in the T2DM group was significantly lower than that of the non-T2DM group both at bas `eline and after the intervention. The proportion of collinsella was lower in the T2DM group (**Figure 6A**). Further analysis of the specific species microbiota showed that there were no differences among groups in the mean proportion of the metabolic-related microbiota, as well as in produces short-chain fatty acids-related microbiota and gut probiotics (**Figures 6B-D**) ylogenetic Investigation of Communities using Reconstruction of Unobserved States (Picrust2) software. The results showed that these metabolism-related pathways consisted of carbohydrate metabolism, amino acid metabolism, transcription, replication, recombination and repair, and other metabolic pathways in the non-T2DM and T2DM group (**Figure 7**).

## 4 Discussion

IGT is closely associated with metabolic disease progression. According to the epidemiological data, about 70% of IGT patients progress to DM within 5 years in China (11). The rapidly growing trend means an urgent need to prevent DM actively. Early dietary modification can prevent the development of diabetes, but it is difficult for individuals to adhere to. The gut microflora plays a crucial role in regulating host metabolism. Changing the composition and/or metabolic activity of gut microflora may contribute to human health. Evidence from human and animal studies suggest that the gut microbiome is a common pathway mediating the therapeutic effects of bariatric surgery, dietary control, and hypoglycemic drug therapy (12–14). Therefore, remodeling the gut microflora may be a new direction for humans to prevent and treat diabetes.

The treatment of metabolic diseases with probiotics is a hot topic in intestinal microbiota research. However, there are fewer studies on probiotics for the prevention and treatment of IGT patients. We previously observed that probiotics supplementation for IGT patients for 2 years did not significantly reduce the risk of IGT conversion to T2DM in the PPDP study (9). In the present study we intend to observe the impact of early and long-term probiotics supplementation on the conversion of diabetes in a longer time (6 years). This is the first long-term prospective study to analyze the efficacy of probiotic administration on glucose metabolism in IGT subjects. The supplementary probiotics in the PPDP study were provided by Bifico (Approval number: S10950032), an over-the-counter capsule consisting of live combined Bifidobacterium longum, Lactobacillus acidophilus and Enterococcus faecalis. It has been reported that Bifidobacterium longum supplementation can attenuate hyperglycemia, improve the antioxidant capacity of the liver, repair intestinal barrier injury, and reduce inflammation in diabetic mice (15). Lactobacillus acidophilus was also reported that can alleviate T2DM by regulating hepatic glucose, lipid metabolism and gut microbiota in mice (16). In addition, it was indicated that Enterococcus faecalis treatment could improve glucose homoeostasis, increased energy expenditure and reduced hepatic steatosis in the db/db mice fed with high fat (17). However, in the present study, after 6 years’ follow up, the Probiotic group showed no significant superiority in preventing the conversion of IGT to T2DM as compared with the Placebo group. Similarly, no significant differences in the diversity and composition of the gut microbiota were observed between the two groups, nor were differences in microbiota observed between groups with or without T2DM. COX regression also showed that probiotics intervention was not affecting IGT conversion to T2DM. Only 30-minute-insulin after glucose loading was the factor affecting the conversion of IGT into T2DM, which indicated that the decrease in islet β -cell function was an important cause of T2DM.

Although the relationship between the gut microbial ecological imbalance and the development of obesity and diabetes is being extensively explored, the conclusions of various studies are different. The results of randomized controlled studies on pregnant women with gestational diabetes or obesity showed that probiotic intervention had no effect on glycemic control, but might improve lipid metabolism (18, 19).In another study of prediabetes adolescents, it was not observed that oral probiotics could improve FBG and HbA1c after 4 months (20). Similarly, in a 24-week probiotic intervention study on adults with prediabetes, the goodness of glycosylated hemoglobin was not observed (21). Our studies are consistent with the conclusion of these studies that probiotics have a limited therapeutic effect on metabolic diseases. However, some studies confirm the beneficial role of gut microbiota in glycemic control and T2DM. Tonucci et al. found that Probiotic consumption improved glycemic control in T2DM subjects (22). The application of a novel probiotic formulation to T2DM showed that the intervention was safe and well tolerated (23). Different probiotic strains, their combinations or the time and duration of intervention may play different roles in the efficacy of the probiotic intervention on glucose control. The limited sample size and subject-to-subject variability suggest that future studies are needed to confirm and extend these observations.

The gut microbiota profile may be related to and responsive to a particular dietary pattern (24). Therefore, supplementation with beneficial microorganisms such as probiotics and their metabolites may alter microbiota distribution and thus affect metabolic parameters (25). However, in this study, gut microbiota analysis results showed no difference in the composition and diversity of the gut microbiota between the T2DM group and the IGT group after two years of probiotic intervention. This may be related to the small fecal sample size selected in this study and the large individual differences of samples within the same group, or it may be the result of functional variation of the strain, indicating that a more precise strategy is required for probiotic therapy. The analysis of specific microbiota showed that compared with the IGT group, the proportion of subdoligranulum, collinsella and monoglobus in the T2DM group decreased after two years of intervention. The occurrence of T2DM may be related to the changes in the composition of intestinal microbiota. Although there is a lack of consensus on which microbiota are significantly changed in T2DM, a common observation has been a decreased abundance of butyrate-producing bacteria with this condition (26). Subdoligranulum and collinsella have been proven to produce butyric acid (27, 28), and a study has shown that the decrease of Monoglobus may be related to insulin resistance and systemic inflammation (29).

There are some limitations in our study. First, this was a small sample size study that enrolled a limited number of patients with IGT. More clinical and laboratory studies using large-size samples and long-term observation are needed to confirm the role of probiotics in developing IGT into DM. Second, the results of the study of Bifico used in this study as a probiotic supplement for Chinese patients are not representative of the effects of other strains on other people or races. Third, the study did not document lifestyle factors, such as diet and exercise, which might have influenced blood sugar outcomes. There is also no recorded family history of T2DM, which is a very strong risk factor for developing T2DM. However, the placebo control designed in this study could compensate for this effect to the greatest extent. To provide preliminary data that could drive more conclusive testing. Therefore, high-quality, large-scale, multicenter randomized controlled trials with longer follow-up are needed to compare safety and efficacy further.

## 5 Conclusions

Nevertheless, the results of this study suggest that supplementation with active probiotics of Bifidobacterium, Lactobacillus acidophilus and Enterococcus faecalis is safe, although it does not reduce the risk of IGT conversion to DM. More clinical and laboratory studies using large samples and long-term observation are needed to explore the effects of different probiotic strains on IGT. This pilot study was designed to provide preliminary data to conduct more conclusive hypothesis testing.

## Data availability statement

The datasets presented in this study can be found in online repositories. The names of the repository/repositories and accession number(s) can be found below: NCBI BioProject [https://www.ncbi.nlm.nih.gov/bioproject/], PRJNA923108.

## Ethics statement

The studies involving human participants were reviewed and approved by the institutional review board of Shanghai East Hospital and was conducted in accordance with the Declaration of Helsinki. The patients/participants provided their written informed consent to participate in this study.

## Author contributions

BF designed the study and oversaw the project implementation. QY conceived and carried out experiments. WH and YT participated in data analyses, interpretation and writing publications. XUL, YY and XIL participated in data collection, data analyses and interpretation and writing publications. All authors were involved in writing the paper and had final approval of the submitted and published versions.
