# Supplementary material for: Probiotics intervention in preventing conversion of impaired glucose tolerance to diabetes: The PPDP follow-on study
Source: Front Endocrinol (Lausanne). 2023 Feb 17;14:1113611. doi: 10.3389/fendo.2023.1113611 (PMC9982119; doi:10.3389/fendo.2023.1113611)
Supplement: Supplementary file 1 [file DataSheet_1.doc]

**Supplementary material 1**

- 1. Microbial diversity analysis
     1. Sample Collection

Fresh fecal samples were collected and stored at -80°C immediately.

- - 1. DNA extraction and PCR amplification

Microbial DNA was extracted from fecal samples using the QIAamp fast DNA stool minikit (Qiagen, CA, USA) according to manufacturer’s protocols. Concentration and purity of the DNA were determined by NanoDrop 2000 spectrophotometer (Thermo Scientific, Wilmington, USA), and DNA quality was examined by agarose gel electrophoresis. The V3-V4 regions of the bacteria’s 16S rRNA gene were amplified by PCR with the primer pairs 338F (5’-ACTCCTACGGGAGGCAGCAG-3’) and 806R (5’-GGACTACHVGGGTWTCTAAT-3’). PCR reactions were performed in triplicate 20 μL mixture containing 4 μL of 5 × FastPfu Buffer, 2 μL of 2.5 mM dNTPs, 0.8 μL of each primer (5 μM), 0.4 μL of FastPfu Polymerase, and 10 ng of template DNA.

- - 1. Illumina MiSeq sequencing

The resulted PCR products were extracted from 2% agarose gels and purified using the AxyPrep DNA Gel Extraction Kit (Axygen Biosciences, Union City, CA, U.S.) according to the manufacturer’s instructions. Purified amplicons were pooled in equimolar and paired-end sequenced (2×250) on an Illumina MiSeq platform according to the standard protocols. The raw reads were deposited into the NCBI Sequence Read Archive (SRA) database (Accession Number: PRJNA923108).

- - 1. Processing of sequencing data

Raw fastq files were demultiplexed, quality-filtered using QIIME (version 1.17) with the following criteria: (i) The 300 bp reads were truncated at any site receiving an average quality score <20 over a 50 bp sliding window, discarding the truncated reads that were shorter than 50bp. (ii) exact barcode matching, 2 nucleotide mismatch in primer matching, reads containing ambiguous characters were removed. (iii) only sequences that overlap longer than 10 bp were assembled according to their overlap sequence. Reads which could not be assembled were discarded.

Operational Units (OTUs) were clustered with 97% similarity cutoff using UPARSE（version 7.1 <http://drive5.com/uparse/>) and chimeric sequences were identified and removed using UCHIME. The taxonomy of each 16S rRNA gene sequence was analyzed by RDP Classifier (http://rdp.cme.msu.edu/) against the silva (SSU115)16S rRNA database using confidence threshold of 70%.
